# Refractive outcome of keratoconus treated by big-bubble deep anterior lamellar keratoplasty in pediatric patients: two-year follow-up comparison between mechanical trephine and femtosecond laser assisted techniques

**DOI:** 10.1186/s40662-018-0127-9

**Published:** 2019-01-07

**Authors:** Luca Buzzonetti, Gianni Petrocelli, Paola Valente, Sergio Petroni, Rosa Parrilla, Giancarlo Iarossi

**Affiliations:** Ophthalmology Department, Bambino Gesù IRCCS Children’s Hospital, Via Torre di Palidoro snc - 00050 Passoscuro, Rome, Italy

**Keywords:** Pediatric keratoplasty, Deep anterior lamellar keratoplasty, Femtosecond laser

## Abstract

**Background:**

To evaluate refractive outcome 24 months after Deep Anterior Lamellar Keratoplasty (DALK) in pediatric patients by comparing results achieved using mechanical trephine and femtosecond laser.

**Methods:**

Twenty eyes of 20 patients affected by keratoconus were evaluated. To perform big-bubble DALK, 10 eyes (Group 1; mean age 11.2 ± 2.2 years) were subjected to the Hessburg-Barron mechanical trephine and the remaining 10 eyes (Group 2; mean age 11.3 ± 3.1 years) to a 150 kHz femtosecond laser that performed mushroom incisions. Preoperative thinnest point in the corneal thickness map and K readings were measured by the Sirius Scheimpflug camera. We also evaluated corrected distance visual acuity (CDVA) as logMAR value using spectacles, spherical equivalent and refractive astigmatism.

**Results:**

Mean preoperative thinnest point and pre- and post-operative K readings did not show significant difference (*P* > 0.05) between the two groups. CDVA, spherical equivalent and refractive astigmatism were respectively, 0.14 ± 0.08 logMAR and 0.13 ± 0.10 logMAR (*P* = 0.8), − 4.2 ± 1.1 D and − 2.8 ± 1.2 D (*P* = 0.03), 4.4 ± 2.0 D and 3.6 ± 1.2 D (*P* = 0.4) in Groups 1 and 2. All DALK procedures were uneventful.

**Conclusion:**

Our findings suggest that femtosecond laser compared to mechanical trephine could significantly reduce the spherical equivalent amount in pediatric big-bubble DALK.

## Background

Deep anterior lamellar keratoplasty (DALK) is indicated for treatment of corneal diseases that do not affect the endothelium. Donor lamella is directly positioned on the Descemet membrane in order to preserve the recipient endothelium and decrease the risk of immunological rejection [1, 2]. Anwar and Teichmann [3] introduced the big-bubble technique in DALK where an air bubble is injected in the deep stroma in an attempt to produce a separation of the Descemet membrane from the overlying anterior cornea thereby providing an easier dissection of the stroma.

In pediatric patients [4, 5], penetrating keratoplasty (PK) represents the current standard procedure also for keratoconus, although the graft failure is reported in approximately 50–60% of cases [4, 5] mainly because of endothelial rejection [6]. To date, there are no reports of pediatric DALK in keratoconus.

Recently the femtosecond solid-state laser [7] was successfully used in corneal transplantation surgery [8]. This technology consists of an infrared Nd:Glass laser beam focused at the desired corneal depth to induce optical breakdown (called photodisruption) without thermal or shockwave damage to the surrounding tissue. Femtosecond laser improves reproducibility and outcomes in corneal lamellar surgery [9–11]. Some authors have investigated the application of femtosecond laser in pediatric surgery [12–14], but long-term follow up have not yet been reported. In this paper we report the refractive outcome 2 years after big-bubble DALK performed in pediatric patients, comparing results achieved using mechanical trephine and femtosecond laser.

## Materials and methods

Twenty patients affected by keratoconus treated with uneventful DALK were enrolled in this retrospective study. A history of atopy with eye rubbing was recorded in 60% of cases. Inclusion criteria were keratoconus progression measured by K readings, progressive corneal thinning and Corrected Distance Visual Acuity (CDVA) decrease. Patients affected by hydrops were excluded. Ten eyes of 10 patients (mean age 11.2 ± 2.2 years; range, 8 to 16 years) underwent DALK using mechanical trephine (Group 1), while 10 eyes of 10 patients (mean age 11.3 ± 3.1 years; range, 7 to 17 years) underwent DALK assisted by femtosecond laser (Group 2). The laser was positioned in the operating room. Four patients of Group 1 and 5 of Group 2 had previously undergone corneal cross linking. All interventions were performed under general anesthesia from April 2013 to May 2015 at the Bambino Gesù Children’s Hospital of Rome, Italy, by one surgeon (L.B.). Informed consent to the procedure was collected for each patient and all experimental investigations followed the guidelines for experimental investigation with human subjects required by the institution. The study adhered to the Tenets of the Declaration of Helsinki.

In Group 1, trephination was performed up to 60% of corneal thickness using the Hessburg-Barron disposable vacuum trephine (Altomed Ltd.), each quarter turn of the device from the zero position producing a cut of about 0.06 mm depth. The Fogla pointed dissector (Bausch & Lomb Storz Ophthalmic) designed to facilitate lamellar dissection of corneal stroma was then used to create a stromal channel for the air injection cannula (the Fogla 27 gauge air injection cannula, Bausch & Lomb Storz Ophthalmic). The donor (8.25 mm diameter) was trephined with a disparity of 0.25 mm. Donor Descemet membrane was peeled off after staining with trypan blue dye.

In Group 2, corneal trephination was performed using the IntraLase 150 kHz femtosecond laser (IntraLase FS Laser, Abbott Medical Optics, Inc.) planning mushroom incisions. This laser requires placement of a suction ring and an applanation lens. After obtaining a proper vacuum seal, we applied the applanation lens to provide a uniform reference plane for the laser. Big-bubble DALK was performed according to the Intrabubble technique [14, 15]. We used the same laser settings for recipient and donor, except for donor outer diameter disparity of 0.25 mm (8.25 mm donor outer diameter). The donor cornea was removed from Optisol storage (Bausch & Lomb), mounted on an artificial anterior chamber (Coronet Patient Artificial Anterior Chamber, Network Medical Products, Ltd.) and then treated under the femtosecond laser. Donor Descemet membrane was peeled off after staining with trypan blue dye.

All patients were maintained on steroid and antibiotic eye drops (0.1% dexamethasone and tobramycin 0.3%) administered every 2 h for 2 weeks. The steroids (0.1% fluorometholone) were tapered over a few months after the procedure to achieve a maintenance dose of once or twice daily.

All grafts were sutured using 4 single 10–0 monofilament nylon sutures and a single 16 bites running suture. Sutures were removed by 8 postoperative months in all patients. Early suture removal was done in cases where sutures were loose, broken, infiltrated, or vascularized. All sutures were usually removed within 5 months in children younger than 8 years and by 8 months in older children.

Preoperative mean thinnest point in the corneal thickness map and K readings were measured by the Sirius Scheimpflug camera (CSO, Firenze, Italy). CDVA analyzed as the logMAR value using spectacles, spherical equivalent and refractive astigmatism calculated by subjective measurement were evaluated and a paired Student *t*-test was used to compare 24 months postoperative data between Groups 1 and 2. *P* < 0.05 was considered statistically significant.

## Results

All DALK procedures were uneventful.

The mean preoperative thinnest point did not show significant difference (*P* > 0.05) in two groups (Group 1, μ = 474 ± 50 μm; Group 2, μ = 470 ± 60 μm) as well as pre- and post-operative K readings values (pre-operative Kmax: Group 1, 60 ± 2 D and Group 2, 58 ± 2.5 D; post-operative Kmax: Group 1, 50 ± 1.5 D and Group 2, 51 ± 2 D).

Table 1 reports preoperative refractive data for both groups. Refractive outcome for each group are summarized in Tables 2 and 3. Two years after surgery, mean postoperative CDVA (logMAR) was 0.14 ± 0.08 and 0.13 ± 0.10 (*P* = 0.8), mean spherical equivalent was − 4.2 ± 1.1 D and − 2.8 ± 1.2 D (*P* = 0.03), and mean refractive astigmatism was 4.4 ± 2.0 D and 3.6 ± 1.2 D (*P* = 0.4), in Groups 1 and 2, respectively (Table 4).

**Table 1:** Preoperative corrected distance visual acuity, spherical equivalent and refractive astigmatism mean value in Groups 1 and 2

	CDVA (logMAR)	Spherical equivalent (D)	Refractive astigmatism (D)
Group 1	0.5 ± 0.1	−12.0 ± 3.1	7.0 ± 2.0
Group 2	0.4 ± 0.2	−10.0 ± 4.2	6.0 ± 1.8
*P* value	0.7	0.9	0.4

*CDVA* = corrected cistance visual acuity; *D* = diopters

The *P* value of paired Student *t*-test was considered significant if it was less than 0.05

**Table 2:** Corrected distance visual acuity, spherical equivalent and refractive astigmatism measured in Group 1 at 24 months after surgery

Patient	Age (years)	CDVA (logMAR)	Spherical equivalent (D)	Refractive astigmatism (D)
1	16	0.2	3.00	9.5
2	12	0.15	−4.50	5.0
3	8	0.15	−4.50	3.0
4	10	0.3	−4.00	3.5
5	11	0.2	−3.50	4.0
6	11	0.04	−5.00	4.0
7	12	0.15	−4.50	4.5
8	9	0.09	−5.00	2.0
9	13	0	−3.25	3.5
10	10	0.15	−2.00	5.5

*CDVA* = corrected cistance visual acuity; *D* = diopters

**Table 3:** Corrected distance visual acuity, spherical equivalent and refractive astigmatism measured in Group 2 at 24 months after surgery

Patient	Age (years)	CDVA (logMAR)	Spherical equivalent (D)	Refractive astigmatism (D)
1	10	0.09	−1.25	2.5
2	17	0	−1.75	3.5
3	15	0.09	−2.00	2.0
4	7	0.3	−2.50	2.5
5	9	0.2	−3.50	4.0
6	10	0.15	−4.00	6.0
7	12	0.09	−2.00	3.0
8	14	0.3	−5.00	5.0
9	9	0.09	−3.50	3.5
10	10	0.04	−2.75	4.5

*CDVA* = corrected cistance visual acuity; *D* = diopters

**Table 4:** Corrected distance visual acuity, spherical equivalent and refractive astigmatism mean value in Groups 1 and 2 at 24 months after surgery

	CDVA (logMAR)	Spherical equivalent (D)	Refractive astigmatism (D)
Group 1	0.14 ± 0.08	−4.2 ± 1.1	4.4 ± 2.0
Group 2	0.13 ± 0.1	−2.8 ± 1.2	3.6 ± 1.3
*P* value	0.8	0.03	0.4

*CDVA* = corrected cistance visual acuity; *D* = diopters

The *P* value of paired Student *t*-test was considered significant if it was less than 0.05

## Discussion

Deep anterior lamellar keratoplasty may offer an advantage to pediatric patients affected by stromal pathologies. Few authors reported indications and outcomes of DALK in children and, in general, this technique is not commonly performed in pediatric patients because of its steep learning curve and surgical complexity [16, 17]. Harding et al. [17] reported outcomes of some DALK with manual dissection performed in pediatric patients affected by partial thickness corneal scarring and mucopolysaccharidoses. Ashar et al. [18] evaluated 26 eyes after DALK (three of them underwent big-bubble technique and 23 layer-by-layer dissection) and observed that DALK is a feasible option in children with stromal corneal pathology.

DALK advantages are well known; it prevents immune rejection, the surgical procedure is extraocular, topical corticosteroids can usually be discontinued earlier and the endothelial cell density is preserved more than in PK. Moreover, in comparison to PK, DALK provides superior resistance to globe rupture after blunt trauma, and sutures can be removed earlier [19, 20].

We have proposed a variant of big-bubble DALK assisted by femtosecond laser [15]. The use of femtosecond laser allows a pre-Descemet lamellar dissection at a predefined corneal depth and the creation of a deep stromal channel 50 μm above the endothelium for introducing a smooth cannula for air injection. The femtosecond laser is accurate in achieving the desired corneal depth and the big-bubble, and could provide good refractive outcome due to the good alignment of donor and recipient. This technique has been successfully applied to children [14].

In this paper, we report refractive outcome 24 months after big-bubble DALK in pediatric patients comparing the Anwar [3] and femtosecond laser [15] assisted techniques. In our clinical practice, we currently perform big-bubble DALK in pediatric patients as well as in adults. Refractive evaluation was performed after suture removal for optical correction and, in order to achieve a more stable postoperative astigmatism in each patient, we stated a 2-year follow up as an appropriate time for comparing refractive outcome between mechanical trephine and femtosecond laser assisted techniques.

Several variables may affect refractive outcome in pediatric DALK. In order to limit them in the comparison between the two big-bubble techniques, we always employed the 8.25 mm donor punch, but in addition, we chose a selection bias by enrolling only patients that underwent an uneventful surgery. We excluded cases (one in Group 1 and two in Group 2) completed by “layer by layer” dissection, and (one in group 1) intraoperative conversion to penetrating keratoplasty.

The small sample size could represent a limitation in the statistical analysis, but it is noteworthy [16, 17] that in general, only few pediatric patients can undergo DALK because of patients’ age as well as preoperative corneal diagnosis.

In this work, we found a statistically significant difference between the two groups only for spherical equivalent values, whereas CDVA and refractive astigmatism did not differ significantly. For these latter two parameters, the difference in two groups was not statistically significant probably due to the small sample size. However, the femtosecond laser group showed less spherical equivalent and postoperative astigmatism. Moreover, in our practice, both methods provide the same percentage of air bubble formation.

The femtosecond laser cutting could in part contribute to the significant spherical equivalent difference. As previously reported [21], since femtosecond laser improves donor-host matching and interlocking, it increases surface contact between donor and recipient, then providing a customized donor-host match. This effect, produced by mushroom geometry, helps the healing process and induces a good biomechanical stability according to Shehadeh-Mashor et al. [11] who observed that the mushroom configuration in DALK for keratoconus combines the advantage of a large anterior surface with reduced surface distortion, providing a discrete wound edge to join the recipient and donor cornea. Instead, mechanical trephination works differently; the transplanting hole is not vertical to the corneal surface and consequently, the donor-host transition becomes less precise, causing more tissue misalignment and optical distortion. Moreover, manual trephination could get neither a uniform incision all around the circle nor an individualized precise cutting, thus inducing an irregular graft geometry and graft–host junction instability [22].

## Conclusions

Our findings suggest that, in pediatric patients, big-bubble technique in DALK assisted by femtosecond laser as compared to mechanical trephine could reduce postoperative spherical equivalent amount, but not improve CDVA and refractive astigmatism.

## References

[1] Shimmura S, Tsubota K (2006). Deep anterior lamellar keratoplasty. Curr Opin Ophthalmol.

[2] Vajpayee RB, Tyagi J, Sharma N, Kumar N, Jhanji V, Titiyal JS (2007). Deep anterior lamellar keratoplasty by big-bubble technique for treatment corneal stromal opacities. Am J Ophthalmol.

[3] Anwar M, Teichmann KD (2002). Big-bubble technique to bare Descemet’s membrane in anterior lamellar keratoplasty. J Cataract Refract Surg.

[4] Hovlykke M, Hjortdal J, Ehlers N, Nielsen K. Clinical results of 40 years of paediatric keratoplasty in a single university eye clinic. Acta Ophthalmol. 2014;92:370–7. 10.1111/aos.12198x 23879323

[5] Huang C, O’Hara M, Mannis MJ (2009). Primary pediatric keratoplasty: indications and outcomes. Cornea.

[6] Aasuri MK, Garg P, Gokhle N, Gupta S (2000). Penetrating keratoplasty in children. Cornea.

[7] Kurtz RM, Liu X, Elner VM, Squier JA, Du D, Mourou GA (1997). Photodisruption in the human cornea as a function of laser pulse width. J Refract Surg.

[8] Farid M, Pirouzian A, Steinert RF (2013). Femtosecond laser keratoplasty. Int Ophthalmol Clin.

[9] Mosca L, Fasciani R, Tamburrelli C, Buzzonetti L, Gruccione L, Mandarà E, et al. Femtosecond laser-assisted lamellar keratoplasty: early results. Cornea. 2008;27:668–72. 10.1097/ICO.0b013e31816736b1 18580258

[10] Buzzonetti L, Petrocelli G, Valente P (2012). Femtosecond laser and big-bubble deep anterior lamellar keratoplasty: a new chance. J Ophthalmol.

[11] Shehadeh-Mashor R, Chan C, Yeung SN, Lichtinger A, Amiran M, Rootman DS (2013). Long-term outcomes of femtosecond laser-assisted mushroom configuration deep anterior lamellar keratoplasty. Cornea.

[12] Agarwal A, Brubaker JW, Mamalis N, Kumar DA, Jacob S, Chinnamuthu S (2009). Femtosecond-assisted lamellar keratoplasty in atypical Avellino corneal dystrophy of Indian origin. Eye Contact Lens.

[13] Buzzonetti L, Petrocelli G, Laborante A (2010). Anterior lamellar keratoplasty assisted by IntraLase™ femtosecond laser in pediatric patient. J Pediatr Ophthalmol Strabismus.

[14] Buzzonetti L, Petrocelli G, Valente P (2012). Big-bubble deep anterior lamellar keratoplasty assisted by femtosecond laser in children. Cornea.

[15] Buzzonetti L, Laborante A, Petrocelli G (2010). Standardized big-bubble technique in deep anterior lamellar keratoplasty assisted by femtosecond laser. J Cataract Refract Surg.

[16] Han DC, Mehta JS, Por YM, Htoon HM, Tan DT (2009). Comparison of outcomes of lamellar keratoplasty and penetrating keratoplasty in keratoconus. Am J Ophthalmol.

[17] Harding SA, Nishal KK, Upponi-Patil A, Fowler DJ (2010). Indications and outcomes of deep anterior lamellar keratoplasty in children. Ophthalmology.

[18] Ashar JN, Pahuja S, Ramappa M, Vaddavalli PK, Chaurasia S, Garg P (2013). Deep anterior lamellar keratoplasty in children. Am J Ophthalmol.

[19] Reinhart WJ, Musch DC, Jacobs DS, Lee WB, Kaufman SC, Shtein RM. Deep anterior lamellar keratoplasty as an alternative to penetrating keratoplasty. A report by the American Academy of Ophthalmology. Ophthalmology. 2011;118:209–18. 10.1016/j.ophtha.2010.11.002 21199711

[20] Keane M, Coster D, Ziaei M, Williams K (2014). Deep anterior lamellar keratoplasty versus penetrating keratoplasty for treating keratoconus. Cochrane Database Syst Rev.

[21] Farid M, Steinert RF (2009). Deep anterior lamellar keratoplasty performed with the femtosecond laser zigzag incision for the treatment of stromal corneal pathology and ectatic disease. J Cataract Refract Surg.

[22] Li S, Wang T, Bian J, Wang F, Han S, Shi W (2016). Precisely controlled side cut in femtosecond laser-assisted deep lamellar keratoplasty for advanced keratoconus. Cornea.

